# Virtual reality cricothyrotomy - a case-control study on gamification in emergency education

**DOI:** 10.1186/s12909-024-05133-7

**Published:** 2024-02-15

**Authors:** I Speck, A Merk, V Burkhardt, Flayyih O, C Huber, A Widder, F Everad, C Offergeld

**Affiliations:** 1 Department of Otorhinolaryngology - Head and Neck Surgery, Faculty of Medicine, Medical Center - University of Freiburg, University of Freiburg, Killianstraße 5, 79106 Freiburg, Germany; 2 Dean’s Office for Human Medicine, Faculty of Medicine, Medical Center - University of Freiburg, University of Freiburg, Breisacher Straße 153, 79110 Freiburg, Germany

**Keywords:** Cricothyrotomy, Gamification, Education, Virtual reality, VR

## Abstract

**Background:**

Cricothyrotomy is an invasive and rare emergency intervention to secure the airway in a “cannot intubate, cannot ventilate” situation. This leads to lack of routine. Cricothyrotomy is performed only hesitantly. Therefore, we aim to improve teaching by including a virtual reality (VR) cricothyrotomy as a learning tool.

**Methods:**

We programmed the VR cricothyrotomy in the C# programming language on the open-source Unity platform. We could include 149 students that we randomly assigned to either a study group (VR cricothyrotomy) or control group (educational video). We asked the study group to subjectively rate the VR cricothyrotomy. To evaluate our intervention (VR cricothyrotomy) we took the time participants needed to perform a cricothyrotomy on a plastic model of a trachea and evaluated the correct procedural steps.

**Results:**

The majority of students that performed the VR simulation agreed that they improved in speed (81%) and procedural steps (92%). All participants completed the cricothyrotomy in 47s ± 16s and reached a total score of 8.7 ± 0.7 of 9 possible points. We saw no significant difference in time needed to perform a cricothyrotomy between study and control group (*p* > 0.05). However, the total score of correct procedural steps was significantly higher in the study group than in the control group (*p* < 0.05).

**Conclusions:**

Virtual reality is an innovative learning tool to improve teaching of emergency procedures. The VR cricothyrotomy subjectively and objectively improved correct procedural steps. Digitized education fills an educational gap between pure haptic experience and theoretical knowledge. This is of great value when focusing on extension of factual knowledge.

**Trial registration:**

DRKS00031736, registered on the 20th April 2023.

**Supplementary Information:**

The online version contains supplementary material available at 10.1186/s12909-024-05133-7.

## Background

The cricothyrotomy is an invasive measure in emergency medicine to secure the airway. If all previous attempts to manage the airway, such as mask ventilation or intubation (“cannot intubate, cannot ventilate”) fail a cricothyrotomy must be performed to secure ventilation [1]. The target structure is the cricothyroid ligament, which connects the thyroid cartilage and the cricoid cartilage.

Since cricothyrotomy is a rare intervention [2] and is only performed as an emergency procedure, it is difficult to teach and learn how to perform this intervention. Cricothyrotomy is performed only hesitantly despite indication, as routine is often lacking and people tend to shy away from surgical interventions [1]. It is therefore even more important to teach and learn cricothyrotomy in the best possible way.

**Fig. 1:**
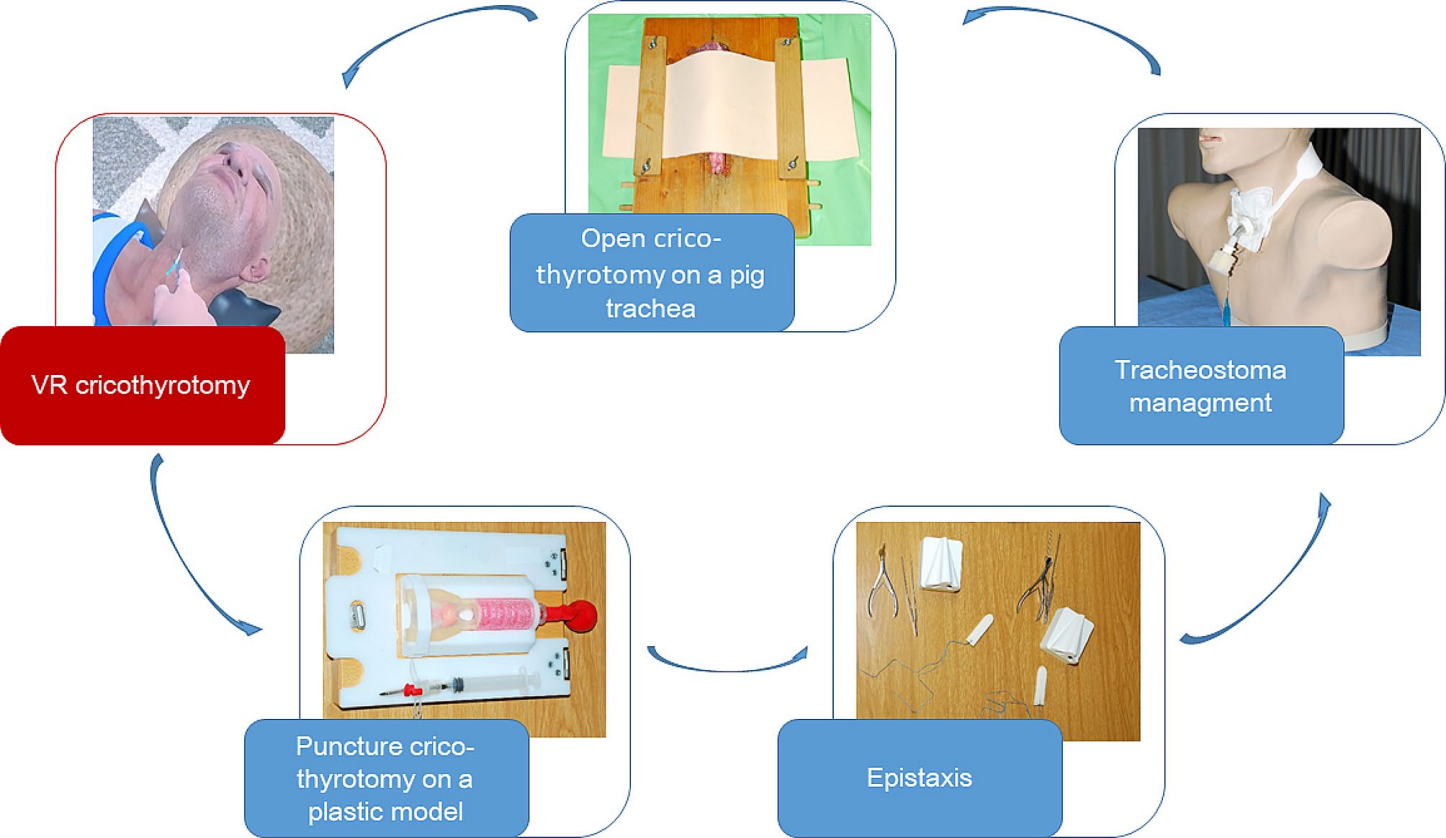
Course of study interventions

At the University of Freiburg, the procedure of open cricothyrotomy has been taught after theoretical introduction on a pig trachea, over which an artificial skin made of sponge rubber (MEYCO® Moosipren, Germany) is stretched (Fig. 1). The puncture cricothyrotomy procedure is practiced on plastic trachea models (VBM Medizintechnik GmBH, Frova Crico-Trainer, Germany) also covered with sponge rubber.

However, these practical exercises only reflect the emergency situation of a cricothyrotomy to a limited extent. It is practiced in a calm atmosphere, without time pressure, and without representation of an affected person. Therefore, we extended the current curriculum by a virtual reality (VR) simulation of a cricothyrotomy. In the VR simulation, the cricothyrotomy is performed on a virtual patient within a time limit of 2 min.

To elevate the learning outcome of the VR simulation we included elements of gamification; the use of elements from the video game industry in a completely different context [3]. However, the goal of gamification is not to offer a game, but to achieve a greater learning effect using strategies from the gaming industry [4].

The number of studies on gamification is still insufficient especially in the field of Otorhinolaryngology and several authors recommend that further studies should be conducted [5, 6]. A recent survey of 2021 from Favier et al. [7] investigated the current use of simulation-based skill training in otolaryngology curricula all over the world. The results regarding the most acquired skills for young otolaryngologist residents yielded tracheotomy (50.4%), emergency cricothyrotomy (48.9%) and rigid bronchoscopy (47.5%). This depicts the need of a VR training of an emergency cricothyrotomy in an otolaryngologist curriculum.

We therefore aimed to examine the subjective and objective implications of a VR cricothyrotomy as an additional teaching tool in the curriculum of students of human medicine.

## Methods

### VR cricothyrotomy

The VR cricothyrotomy was programmed in the C# programming language on the open-source Unity platform (https://unity.com/download). The virtual environment is designed through the Blender program (https://www.blender.org/features/), which is an open-source program for three-dimensional objects, through which many three-dimensional models can be designed in the virtual world (e.g. medical tools, healthcare rooms, etc…). Using the open-source Steam VR extension we created the system for holding 3D objects. In this scenario, a virtual patient is essential, and therefore we applied the makehuman program (http://www.makehumancommunity.org/). “Make human community” is an open-source program in which virtual humans of any age or shape can be designed in a very detailed way. The virtual patient was exported to the Blender program. Additionally, we implemented a point system rating the intervention of the participant during the VR cricothyrotomy.

**Fig. 2:**
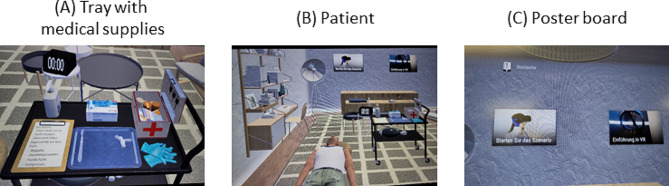
Virtual reality room

During the VR cricothyrotomy scenario the participants were located in a VR room that contained a tray with medical supplies (Fig. 2A), a patient in a “cannot intubate, cannot ventilate” situation (Fig. 2B), and a poster board to control the scenario (Fig. 2C).

The participants have 2 min time to perform the following steps: (A) palpation of the throat, (B) vertical skin incision, (C) horizontal severance of the cricothyroid ligament, (D) keep the trachea open with the handle of the scalpel, (E) insertion of endotracheal tube, (F) ventilation (Supplement 1).

The total score was 100 points. Errors in the cricothyrotomy procedure were punished with loss of points: -10 points for hand disinfection, -10 points for using gloves, -10 points for reading patient history, -10 points for horizontal skin incision, -30 points for vertical incision of the cricothyroid ligament, and − 40 points for failing to keep the trachea open with the handle of the scalpel.

### Participants and study interventions

**Fig. 3:**
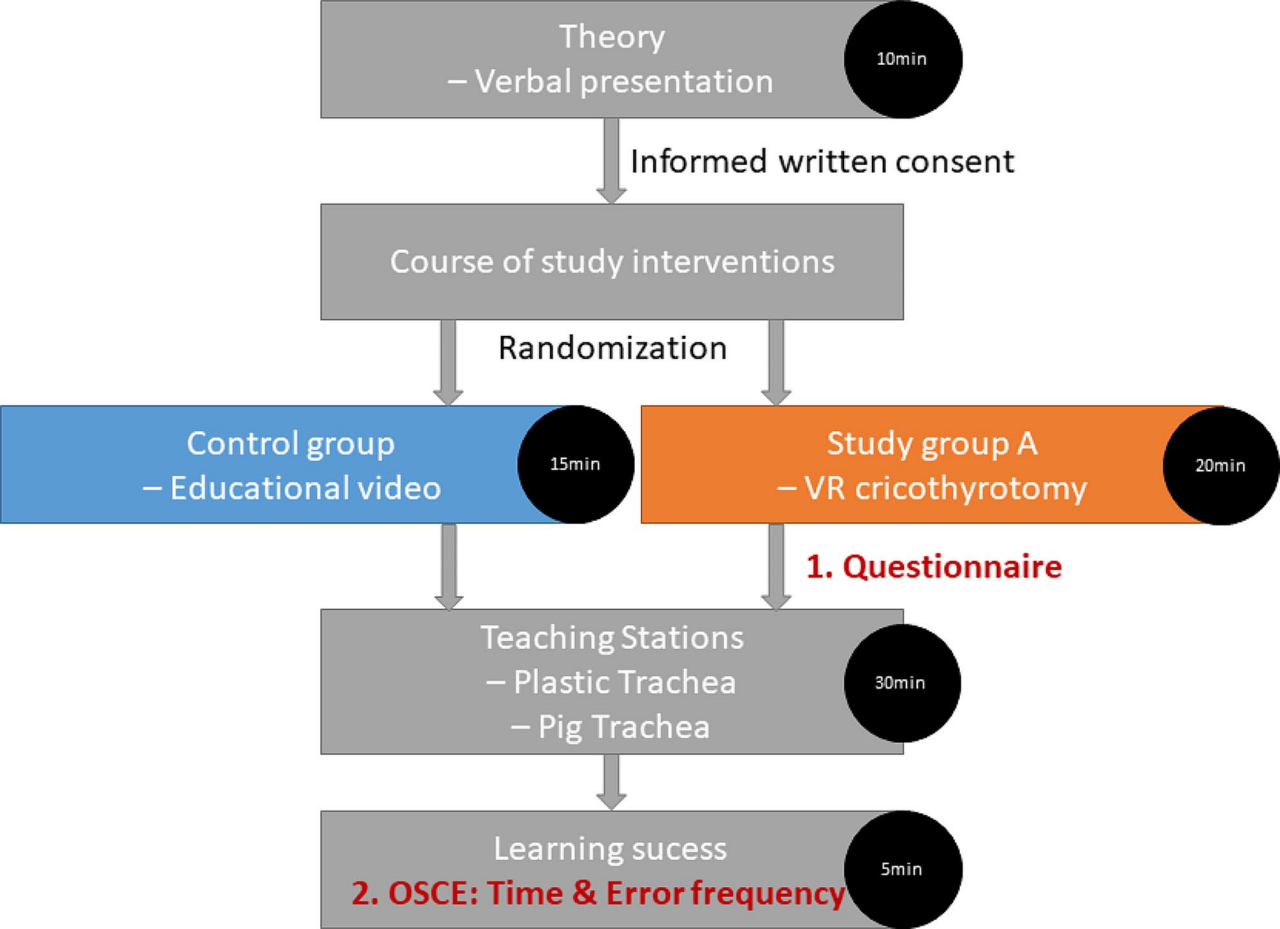
Course of study interventions for study group and control group

We could include 149 students of human medicine of 178 students participating in the summer term 2023. The included students all studied in the 4th medical school year. Additional information, like age or gender, were not collected. All students underwent the educational stations concerning open cricothyrotomy, puncture cricothyrotomy, tracheostomy management and epistaxis (Fig. 1). The participants were randomly assigned to either a study group or control group (Fig. 3).

The study group was asked to evaluate the VR cricothyrotomy with a questionnaire based on the Münster questionnaire for evaluation - additional module role-playing games (Table 1) [8]. The control group watched an educational video including the following information: (1) indication of cricothyrotomy, (2) steps of cricothyrotomy, (3) possible sources of error, and (4) videos of VR cricothyrotomy.

**Table 1:** Questionnaire based on the Münster questionnaire for evaluation - additional module role-playing games [8]

	Very strongly disagree	Strongly disagree	Disagree	Neutral	Agree	Strongly agree	Very strongly agree	N/A
**Q1**: I was able to transfer my theoretical knowledge of performing a cricothyrotomy to the VR simulation.	1	2	3	4	5	6	7	0
**Q2**: My tasks in the VR simulation were clear to me.	1	2	3	4	5	6	7	0
**Q3**: The goals of the VR simulation were made transparent by the instructor(s).	1	2	3	4	5	6	7	0
**Q4**: I was able to practice the procedure and speed of a cricothyrotomy through the VR simulation.	1	2	3	4	5	6	7	0
**Q5**: I have improved my speed through VR simulation.	1	2	3	4	5	6	7	0
**Q6**: I have improved my procedure through the VR simulation.	1	2	3	4	5	6	7	0
**Q7**: The VR simulation met my expectations.	1	2	3	4	5	6	7	0
**Q8**: I found the VR simulation to be unrealistic.	1	2	3	4	5	6	7	0
**Q9**: The instructor(s) gave me useful feedback on my performance in the VR simulation.	1	2	3	4	5	6	7	0
**Q10**: Does gaming experience exist?	Yes				No			
Additional comments								

Both groups were tested on puncture cricothyrotomy and open cricothyrotomy two days after intervention (VR simulation or educational video). The puncture cricothyrotomy was performed on the plastic model of a trachea (VBM Medizintechnik GmBH, Frova Crico-Trainer, Germany) covered with a beige foam rubber (MEYCO® Moosipren, Germany) used during training. We took the time participants needed to perform the puncture cricothyrotomy and evaluated the correct procedural steps. Participants were asked to describe the steps performed while doing them. Every correctly performed step equalled one point: (1) choosing the puncture set, (2) palpation of the throat, (3) identification of the thyroid cartilage, (4) identification of the cricoid cartilage, (5) identification of the cricothyroid ligament, (6) puncture of the trachea, and (7) ventilation. The open cricothyrotomy was simulated on a laminated sheet of paper showing the larynx. The participants were instructed to draw the alignment of the skin incision (vertical) and the severance of the cricothyroid ligament (horizontal). Each correct alignment was awarded with one point. The total score from both assignments was 9 points.

### Statistical analysis

We performed statistical data analysis with the statistics program Gnu R (Version 2022.12.0). We applied the Shapiro-Wilk test to determine whether the data has been drawn from a normally distributed population. As a normal distribution was not present, we used Wilcoxon signed rank tests. To investigate the objective benefit we compared the total score and time between participants in the study and control group. In addition, we calculated the correlation between time and subjective improvement of time (Q5) and total score and subjective improvement of procedure (Q6) as well as transfer of theoretical knowledge (Q1). To validate the impact of gaming experience we compared time and total score between subjects with and without gaming experience, using Wilcoxon signed rank tests.

## Results

### Questionnaire

**Fig. 4:**
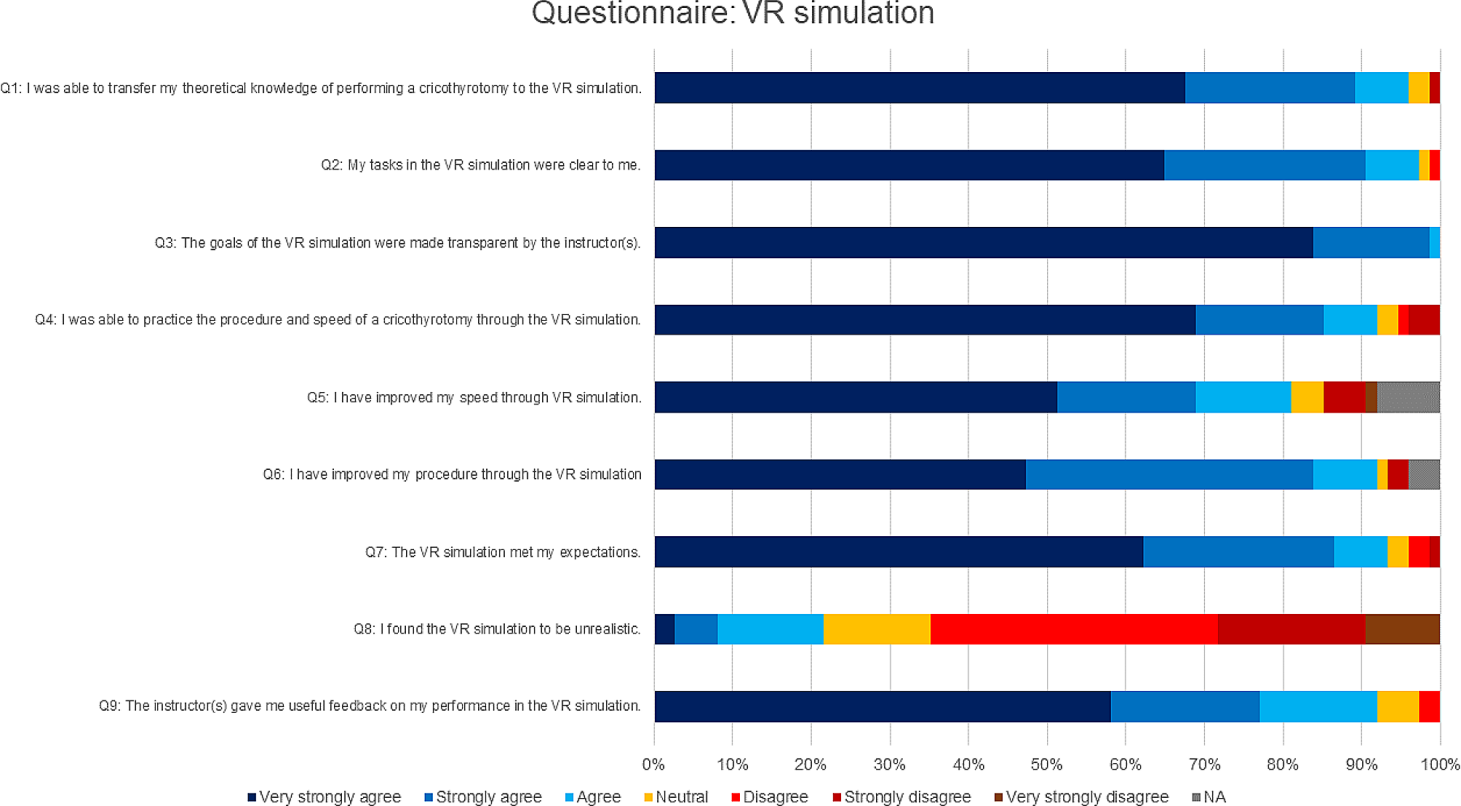
Evaluation of VR simulation

The majority of students (Q1: 96%, *n* = 71) that performed the VR simulation very strongly agreed, strongly agreed or agreed with the statement: “I was able to transfer my theoretical knowledge of performing a cricothyrotomy to the VR simulation” (Fig. 4). Most students very strongly agreed, strongly agreed or agreed that the task was clear (Q2: 97%, *n* = 72), that the goals were made clear (Q3: 100%, *n* = 74) and that the feedback was useful (Q9: 92%, *n* = 68). Improvement in speed (Q5: 81%, *n* = 60) and procedure (Q6: 92%, *n* = 68) was subjectively achieved by most students. Approximately two thirds of the students thought that the VR simulation was realistic (Q8: 65%, *n* = 48), whereas approximately a quarter of the students perceived the VR simulation as unrealistic (Q8: 22%, *n* = 16).

### Objective results

Our participants completed the puncture cricothyrotomy in 47s ± 16s and reached a total score of 8.7 ± 0.7 of 9 possible points.

The study group needed less time to perform the puncture cricothyrotomy than the control group (study group: 44s ± 15s vs. control group: 50s ± 17s). This difference only reached trend levels (*p* = 0.09). The time was not correlated with the subjective improvement of time through the VR simulation (Q5) in the study group (*p* > 0.05, r^2^=-0.07).

**Fig. 5:**
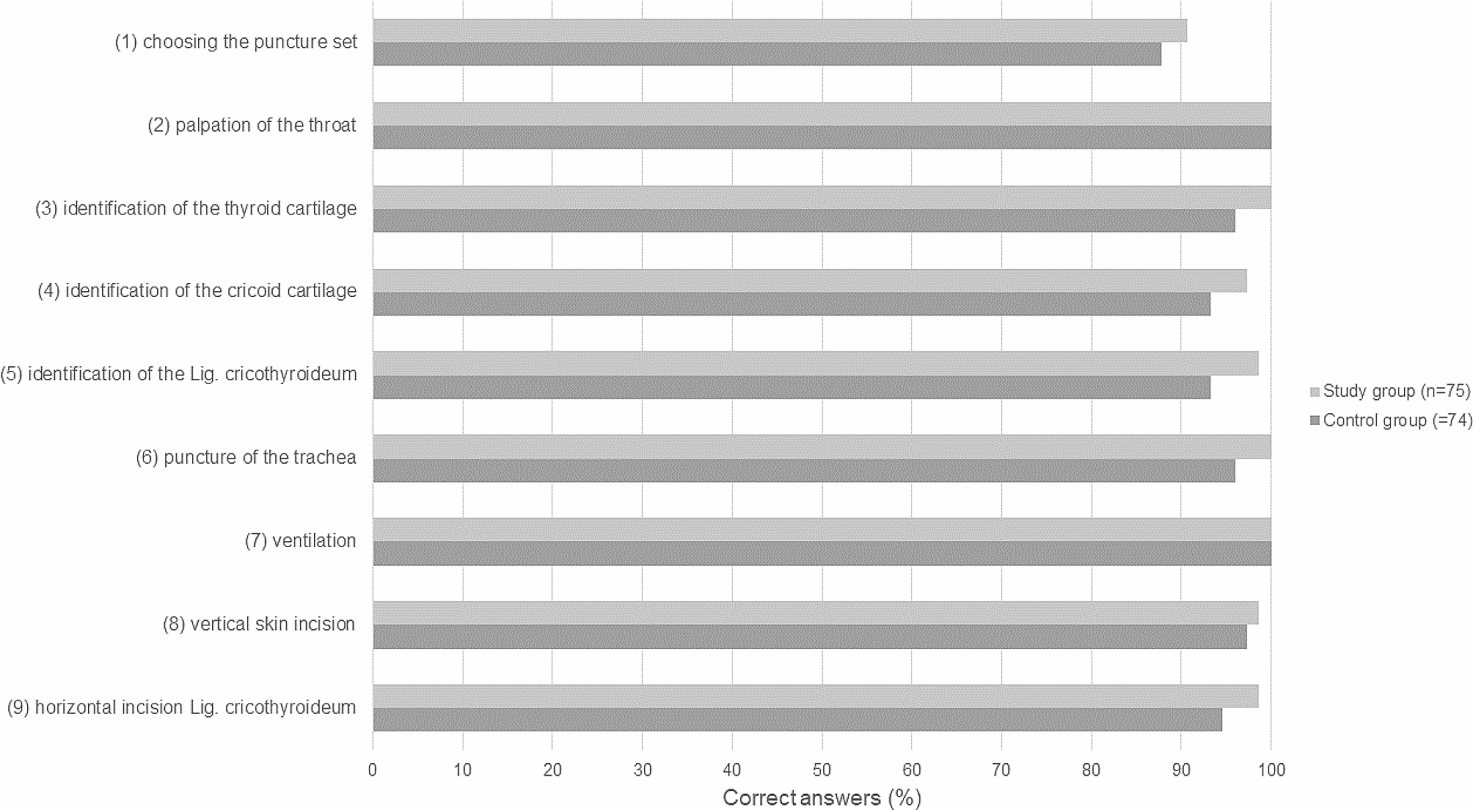
Total scores of students in the study group and control group

The study group reached a higher total score in the cricothyrotomy than the control group (study group: 8.9 ± 0.4 vs. control group: 8.5 ± 0.9). The difference reached statistical significance (*p* = 0.04, Fig. 5). Students of the study group outperformed students of the control group in each category. The score differed on a trend level in the categories: (3) identification of the thyroid cartilage (*p* = 0.08), (5) identification of the Lig. cricothyroideum (*p* = 0.09), and (6) puncture of the trachea (*p* = 0.08). The total score was not significantly correlated with the subjective improvement in procedure (Q6: *p* > 0.05 r^2^=-0.14) or transfer of theoretical knowledge (Q1: *p* > 0.05 r^2^=-0.11).

Prior gaming experience did not significantly affect time or total score (both *p* > 0.05).

## Discussion

In the present study, we investigated the subjective and objective implications of introducing a VR cricothyrotomy into our curriculum for students of human medicine.

Introducing VR simulation of cricothyrotomy, we saw a significant increase in correct procedural steps performed by the students that received VR cricothyrotomy training compared to students that only watched an educational video (control group). The students in the study group were also faster than the students in the control group – however, this difference did not reach significance. Our results are similar to Sankaranarayanan et al. [9], who trained a group of 10 medical students with the VAST-CCT (virtual airway skills trainer - cricothyroidotomy) - a virtual reality simulator for training in critical airways. After two weeks of training both groups performed the procedure on a TraumaMan (Simulab, Seattle, Washington). Students in the study group showed faster and better performance in cricothyrotomy compared with the control group [9]. After training with a VAST-CCT the time improved from 193.2 to 42.1 s. These findings match with ours regarding a better performance of the study group during the puncture cricothyrotomy. However, we did not see a significantly faster performance of the procedure, merely a trend in favor of the study group (44s vs. 50s). This could be explained by the smaller study group (*n* = 10 vs. *n* = 75), the longer training set up of two weeks and the comparison of pre- and post-intervention times in the study of Sankaranarayanan et al. [9]. In the present study we did not test the time on a model of an open cricothyrotomy but on a plastic model of puncture cricothyrotomy. We only tested the open cricothyrotomy on laminated paper limiting our value of the present study. In future studies we plan to perform the testing on either an plastic model for open cricothyrotomy or pig trachea. Additionally the control group of the present study was shown an educational video whereas the control group in the study of Sankaranarayanan et al. [9] received no intervention. It is to further note, that both our study and our control group are closer to the post-intervention time of (42.1 vs. 44/50s) than the pre-intervention time (139s).

A disadvantage of the VR simulation is the missing haptic input. Therefore, we see the VR simulation as an addition to manikin-based training and training on a pig trachea. Takayesu et al. [10] showed that cadaver-based training is superior to training on manikins and can reproduce difficult airway situations. Unfortunately, training on cadavers for more than 150 students per semester is not feasible. Therefore, our curriculum only included training on pig trachea.

In our evaluation, the majority of students (very strongly) agreed that the VR simulation improved their speed and procedure of the cricothyrotomy and helped them to transfer theoretical knowledge. This result is in agreement with a survey on 217 medical students in the USA, of which 80% were convinced that video games can have a learning effect and 77% even stated that they would like to use a “multiplayer online healthcare simulation” [11] in their free time if they could achieve a personal goal. VR is a valuable addition to the field of medical training because it offers a safe and standardized environment [5, 12, 13].

Gaming elements included in VR training should promote and consolidate the transfer of knowledge. Table 2 describes the gaming elements used in the present study and gives reasons for gaming elements not applied in the present study.

**Table 2:** Application of gaming elements in the present study

Gaming element	Included	Excluced	Reasoning
Theoretical questions		x	We excluded theoretical questions to focus on the particle aspect of the exercise.
Point system	x		
Progress bar		x	Since our simulation is performed separately each run, progress is not assessed.
Feedback	x		
Awards		x	Since our simulation is performed separately each run, awards were not given.
Social interaction		x	We included no player-to-player communication, since only one student performed the VR simulation at a time.
Story line	x		

Gaming elements were applied from the review of Westenhaver et al. [6].

We included a point system to give feedback to the VR cricothyrotomy performed by the participant. Additionally, a short storyline was included. As we focused on the particle aspect of the exercise, we did not include theoretical questions in the VR simulation. Another reason to exclude theoretical information is that the VR simulation was integrated into an overall curriculum that contains theoretical background of the cricothyrotomy. If a VR simulation stands alone theoretical information could be included. The VR simulation can be used repeatedly to improve the cricothyrotomy. In this scenario, a progress bar and awards should be included to improve feedback and therefore the learning outcome of the participants. Comparing the time needed in each run could serve as a progress bar of sorts and could also result in an award for the best time achieved. The comparison between participants in form of social interaction could also improve competitiveness between participants and therefore improve the learning experience. However, an overload of gaming element could also distract form the learning outcome. Including more gaming elements, in future studies can show us the importance of each gaming element to the learning outcome.

## Conclusions

Virtual reality is an innovative learning tool to improve teaching of emergency procedures. In our study, which matches investigations of others. The VR cricothyrotomy subjectively and objectively improved correct procedural steps. Digitized education fills an educational gap between pure haptic experience and theoretical knowledge. This makes VR a powerful tool to improve traditional teaching methods and increase student motivation and learning outcomes.

### Electronic supplementary material

Below is the link to the electronic supplementary material.

Supplementary Material 1

## Data Availability

The datasets analysed during the current study are available from the corresponding author on reasonable request.

## Abbreviations

VR: Virtual reality
