# Supplementary figures and images for: Virtual reality cricothyrotomy - a case-control study on gamification in emergency education
Source: BMC Med Educ. 2024 Feb 15;24:148. doi: 10.1186/s12909-024-05133-7 (PMC10868043; doi:10.1186/s12909-024-05133-7)

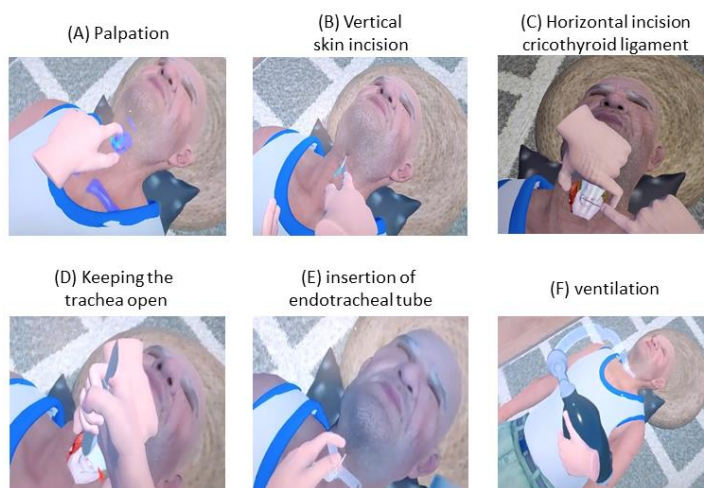

**Supplement 1** *Steps of the VR cricothyrotomy scenario*

Supplement: Supplementary file 1 — Supplementary Material 1 [file 12909_2024_5133_MOESM1_ESM.pdf]
